# *MMP9* polymorphism is associated with susceptibility to non-traumatic osteonecrosis of femoral head in a Chinese Han population

**DOI:** 10.18632/oncotarget.20463

**Published:** 2017-08-24

**Authors:** Yuan Liu, Yanfei Jia, Yuju Cao, Yan Zhao, Jieli Du, Feimeng An, Yuxin Qi, Xue Feng, Tianbo Jin, Jianping Shi, Jianzhong Wang

**Affiliations:** ^1^ Inner Mongolia Medical University, Hohhot, Inner Mongolia, China; ^2^ Department of Orthopedics and Traumatology, The Second Affiliated Hospital of Inner Mongolia University, Hohhot, Inner Mongolia, China; ^3^ Zhengzhou Traditional Chinese Medicine Traumatology Hospital, Zhengzhou, Henan, China; ^4^ College of Life Sciences, Inner Mongolia University, Hohhot, Inner Mongolia, China; ^5^ The College of Life Sciences Northwest University, Xi’an, Shaanxi, China; ^6^ Department of TCM Diagnoses, Inner Mongolia Medical University, Hohhot, Inner Mongolia, China

**Keywords:** MMP9, MMP2, non-traumatic osteonecrosis of the femoral head, single nucleotide polymorphisms, association study

## Abstract

Non-traumatic osteonecrosis of femoral head (ONFH) is an orthopedic refractory disease with escalating morbidity in Chinese Han population. In our case-control study, we examined eight previously identified *MMP9* single-nucleotide polymorphisms (SNPs) in 585 non-traumatic ONFH patients and 507 healthy individuals from northern China to determine whether these SNPs associated with the risk of developing non-traumatic ONFH. Genetic model and haplotype analyses were used to evaluate the association between SNPs and non-traumatic ONFH. *MMP9* rs2274755 (OR, 0.740; 95% CI, 0.578-0.949; p = 0.017) was associated with a reduced risk of non-traumatic ONFH. After adjusting for age and gender, the logistic regression results showed that rs2274755 associated with a lower risk of non-traumatic ONFH in the dominant (OR=0.71, 95% CI: 0.54-0.94, p=0.016), overdominant (OR=0.73, 95% CI: 0.55-0.96, p=0.026) and log-additive (OR=0.74740; 95% CI, 0.578-0.949; p=0.017) models. In addition, the “TGC” haplotype of rs2274755 was associated with a 0.79-fold decrease in risk while the “CTC” haplotype associated with a 0.65-fold decrease risk of the non-traumatic ONFH. These results provide evidence that the *MMP9* SNP at the rs2274755 locus is associated with a decreased risk of non-traumatic ONFH in a Chinese Han population.

## INTRODUCTION

Non-traumatic osteonecrosis of the femoral head (ONFH) is a painful and progressive disorder of the hip joint, mainly affecting middle aged individuals 30-50 years old. Genetic polymorphisms are gene variations found in about 1% of the general population, where they influence protein translation and the expression of related genes to contribute to disease susceptibility [1]. It is believed that genetic polymorphisms may be crucially involved in non-traumatic ONFH [2–4].

Matrix metalloproteinases (MMPs) are a family of 23 Zn^2+^-dependent endopeptidases. Their primary activities involve hydrolysis of the protein components of connective tissues such as extracellular matrix (ECM) and basement membranes [5]. MMPs can be subdivided into matrilysins, stromelysins, gelatinases, and collagenases [6]. The actions of MMPs in pathology can be grouped into three types: (1) destruction of tissue (2) fibrosis (3) matrix weakening [6]. MMP-2 (gelatinase A) and MMP-9 (gelatinase B) constitute the gelatinase subgroup [7]. Within bone, bone marrow mesenchymal stem cells differentiate into osteoclasts and osteoblasts. Balanced bone resorption by the osteoclasts and bone formation by the osteoblasts sustains skeletal homeostasis. Both MMP-2 and -9 are produced by the osteoblasts, and MMP-9 is additionally produced by osteoclasts [8]. Among the substrates of these two enzymes are type I collagen and aggrecan, which are components of cartilage [9]. In addition, collagen type I is a major component of the bone ECM, and its loss correlates with bone resorption [10, 11]. It has therefore been suggested that MMP-2 and -9 may be associated with non-traumatic ONFH.

MMP-2 is the most widely expressed MMP [12]. It has been detected in nearly all tissues and cells. By contrast, expression of MMP-9 is limited to macrophages, monocytes, keratinocytes, and polymorphonuclear cells [12]. During normal bone remodeling, the activities of MMP-9 contribute to the regulation of apoptosis of hypertrophic chondrocytes and growth plate angiogenesis [13, 14]. Notably, expression of both MMP-9 and MMP-2 is enhanced in the joints of patients with rheumatoid arthritis [9, 15], while MMP-9 is overproduced in the osteoarthritic bone tissue [12, 16]. Similarly, levels of MMP-2 and protein are up-regulated in patients with non-traumatic ONFH, though the precise functions neither enzyme has yet to be defined.

In previous case-control studies, we genotyped eight *MMP9* and *MMP2* single-nucleotide polymorphisms (SNPs; rs3918249, rs2274755, rs3918254, rs243832, rs1053605, rs243849, rs243847, rs7201) that are associated with rheumatoid arthritis [9], glaucoma [17], asthma [6], thoracic aortic dissection [18], steroid-induced osteonecrosis [19], myopia, refractive error, ocular biometric measures [20], psoriasis vulgaris [21], endometriosis [22], obesity [23], and alcohol-induced osteonecrosis of the femoral head [24]. The aim of the present study was to identify the associations between these eight SNPs and the susceptibility to non-traumatic ONFH in a Chinese Han population.

## RESULTS

We designed a case-control study examining the potential association between *MMP2* and *MMP9* polymorphism and non-traumatic ONFH in 585 non-traumatic ONFH patients (472 Male, 113 Female) and 507 healthy controls (111 female, 396 male). Gender and age distributions for ONFH patients and controls are shown in Table 1. The cases and controls were matched with respect to gender (p = 0.293) and were adjusted based on age. Primers and PCR product sequences are shown in Table 2.

**Table 1:** Characteristics of cases and controls in male individuals

Variable	Cases	Controls	*p* Value
	n=585	n=507	
Alcohol	285		
Steroids	300		
Sex			0.293^a^
Male	472(80. 7%)	396(78.1%)	
Female	113(19.3%)	111(21.9%)	
Age, year (mean ± SD)	42.61±12.951	47.43±9.739	<0.001^b^

*p* ≤ 0.05 indicates statistical significance.

^a^*p* Two-sided Chi-squared test.

^b^*p* Independent samples t test.

**Table 2:** Primers used for this study

SNP ID	1st-PCR primer sequences	2nd-PCR primer sequences	UEP sequences
rs3918249	ACGTTGGATGAAGCACTGGTGTCTGGAAAG	ACGTTGGATGGATTACAAGTGTGAGCCGTC	gaaGTCATGCCCAGCAGGGACTA
rs2274755	ACGTTGGATGGGGAGAGAATGAAGGGAATC	ACGTTGGATGTTCGACGATGACGAGTTGTG	gCTGGGCAAGGGCGTCGGT
rs3918254	ACGTTGGATGTCTTCGGCTTCTGCCCGAC	ACGTTGGATGCAATACATGATGAGAGGGCG	CTGGTAGACAGGGTGGA
rs1053605	ACGTTGGATGCGTAGCTGCTCCATAAATAG	ACGTTGGATGACAGAGAGAATTTCAGTCCG	gaCGGTAAGCAATGTAATTCATTTCA
rs243849	ACGTTGGATGCTCAAAGTTGTAGGTGGTGG	ACGTTGGATGAAGGAGTACAACAGCTGCAC	AACAGCTGCACTGATAC
rs243847	ACGTTGGATGTACCTTGGTCAGGGCAGAAG	ACGTTGGATGAGTGACGGAAAGATGTGGTG	ACAGCCAACTACGATGA
rs243832	ACGTTGGATGAAGACAAGAGCAGTGACCCC	ACGTTGGATGCCAAAATCAGACCCTGGTAG	ccTGCTGCTACTCACCTCC
rs7201	ACGTTGGATGTCCAATCCCACCAACCCTCA	ACGTTGGATGGCAGGGCTGCGTTGAAAATA	aAGGGCTGCGTTGAAAATATCAAAG

All eight *MMP2* and *MMP9* SNPs were in Hardy-Weinberg equilibrium (p < 0.05). We found that *MMP*9 rs2274755 (OR, 0.740; 95% CI, 0.578-0.949; p = 0.017) was associated with a decreased risk of non-traumatic ONFH (Table 3). The association of *MMP9* SNPs with the risk of non-traumatic ONFH was tested in dominant, recessive, codominant, overdominant, and log-additive models (Table 4). The rs2274755 polymorphism in *MMP9* conferred a protective effect against non-traumatic ONFH in the dominant (OR = 0.71, 95% CI: 0.54-0.94, p = 0.016), overdominant (OR = 0.73, 95% CI: 0.55-0.96, p = 0.026) and log-additive (OR = 0.74, 95% CI: 0.57-0.95, p = 0.017) models. After adjusting for age and gender, our analyses showed the association between rs2274755 an non-traumatic ONFH conferred a protective effect in the codominant “G/T” model (OR = 0.70, 95% CI: 0.52-0.93, p = 0.040), the codominant “T/T” model (OR = 0.65, 95% CI: 0.25-1.68, p = 0.040), the dominant model (OR = 0.70, 95% CI: 0.52-0.92, p = 0.011), the overdominant model (OR = 0.71, 95% CI: 0.53-0.94, p = 0.018), and the log-additive model (OR = 0.72, 95% CI: 0.56-0.94, p = 0.013).

**Table 3:** Allele frequencies in cases and controls and odds ratio estimates for non-traumatic ONFH

SNP	Gene	Locus	Alleles (A/B)	MAF		HWE *p*^*a*^ value	ORs	95%CI	*p*^*b*^ Value
				Case	Control				
rs3918249	*MMP9*	20q13.12	T/C	0.297	0.322	0.613	0.886	0.739-1.062	0.191
rs2274755	*MMP9*	20q13.12	T/G	0.116	0.151	0.729	0.740	0.578-0.949	0.017*
rs3918254	*MMP9*	20q13.12	T/C	0.203	0.187	0.664	1.102	0.891-1.363	0.372
rs1053605	*MMP2*	16q12.2	T/C	0.112	0.129	0.843	0.850	0.656-1.100	0.217
rs243849	*MMP2*	16q12.2	T/C	0.191	0.167	1.00	1.177	0.945-1.468	0.146
rs243847	*MMP2*	16q12.2	C/T	0.415	0.405	0.117	1.042	0.879-1.237	0.634
rs243832	*MMP2*	16q12.2	C/G	0.362	0.382	0.347	0.921	0.774-1.096	0.353
rs7201	*MMP2*	16q12.2	C/A	0.245	0.255	0.349	0.950	0.782-1.154	0.603

SNP: single nucleotide polymorphism, HWE: Hardy-Weinberg equilibrium, OR: odds ratio, 95% CI: 95% confidence interval, MAF: minor allele frequency.

^a^*p* was calculated by fisher's exact test.

^b^*p* was calculated by Pearson Chi-squared test.

A/B=minor/major alleles.

The SNPs were excluded at HWE *P* level of 5%.

**Table 4:** Genotypic model analysis of relationship between rs2274755 and ONFH risk

Model	Genotype	Control	Case	Without adjustment		With adjustment		AIC	BIC
				OR (95% CI)	*p*	OR (95% CI)	*P*		
**Codominant**	G/G	364 (71.8%)	457 (78.1%)	1	0.053	1	0.04*	1508.4	1523.4
	G/T	133 (26.2%)	120 (20.5%)	0.72 (0.54-0.95)		0.70 (0.52-0.93)*			
	T/T	10 (2%)	8 (1.4%)	0.64 (0.25-1.63)		0.65 (0.25-1.68)			
**Dominant**	G/G	364 (71.8%)	457 (78.1%)	1	0.016*	1	0.011*	1506.4	1516.4
	G/T-T/T	143 (28.2%)	128 (21.9%)	0.71 (0.54-0.94)		0.70 (0.52-0.92)*			
**Recessive**	G/G-G/T	497 (98%)	577 (98.6%)	1	0.43	1	0.47	1511.6	1521.6
	T/T	10 (2%)	8 (1.4%)	0.69 (0.27-1.76)		0.70 (0.27-1.83)			
**Overdominant**	G/G-T/T	374 (73.8%)	465 (79.5%)	1	0.026*	1	0.018*	1507.3	1517.3
	G/T	133 (26.2%)	120 (20.5%)	0.73 (0.55-0.96)		0.71 (0.53-0.94)*			
**Log-additive**	—	—	—	0.74 (0.57-0.95)	0.017*	0.72 (0.56-0.94)*	0.013*	1506.5	1516.5

p* ≤ 0.05 indicates statistical significance.

*p* values were calculated by Wald test by unconditional logistic regression adjusted for age and gender.

AIC = Akaike Information Criterion.

BIC = Bayesian Information Criterion.

**Figure 1:**
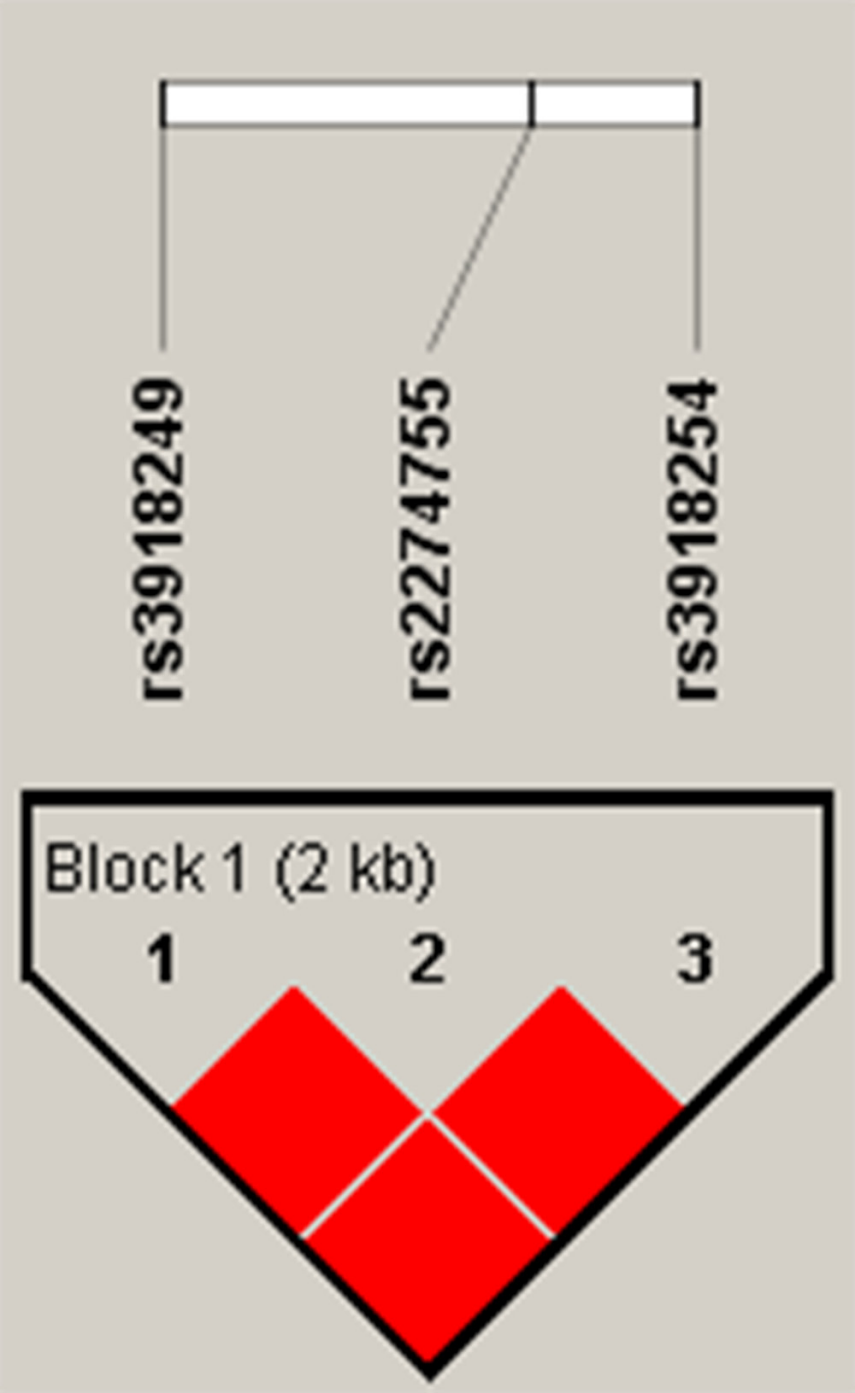
Linkage disequilibrium (LD) plots containing three SNPs from MMP9 Red squares display statistically significant associations between a pair of SNPs, as measured by D’; darker shades of red indicate higher D’.

In addition, the haplotype frequencies of rs3918249, rs2274755 and rs3918254 exhibited an association with non-traumatic ONFH risk “TGC” (Freq = 0.309, OR = 0.79, p = 0.028) and “CTC” (Freq = 0.132, OR = 0.65, p = 0.003). Moreover, the candidate SNP in *MMP*9 showed a strong linkage in the Haplotype analysis (Figure 1). The results of the association between the *MMP*9 haplotype and the risk of non-traumatic ONFH are listed in Table 5. Haplotype “TGC” (OR 0.79; 95 % CI 0.64 - 0.97; p = 0.028) and “CTC” (OR 0.65; 95 % CI 0.49 - 0.86; p = 0.0027) were found to be associated with a decreased risk of non-traumatic ONFH after adjusting the figures based on age and gender. We found no associations between the *MMP*2 polymorphisms and the risk of non-traumatic ONFH.

**Table 5:** The haplotype frequencies of MMP9 polymorphisms and their association with non-traumatic ONFH risk

Haplotype			Freq	OR^a^ (95% CI)	OR^b^(95% CI)	*p*^a^	*p*^b^
rs3918249	rs2274755	rs3918254					
9	5	4					
C	G	C	0.3636	1	1	—	—
T	G	C	0.3086	0.81 (0.66 - 1.00)	0.79 (0.64 - 0.97)	0.047*	0.028*
C	G	T	0.1955	0.96 (0.75 - 1.22)	0.92 (0.72 - 1.18)	0.72	0.52
C	T	C	0.1323	0.68 (0.52 - 0.89)	0.65 (0.49 - 0.86)	0.0051	0.0027*

*p ≤ 0.05 indicates statistical significance.

a= crude analysis data.

b= adjusted by Gender and Age.

## DISCUSSION

Genetic research has provided new insight into non-traumatic ONFH. Several SNPs reportedly associate with the susceptibility to non-traumatic ONFH [25]. In the present study, we showed that genetic variation in the *MMP9* promoter associates with non-traumatic ONFH in a Chinese Han population. Our main findings suggest *MMP9* SNP rs2274755 associates with a decreased risk of non-traumatic ONFH. Haplotype analysis revealed three blocks in rs3918249, rs2274755, and rs3918254 that showed the “TGC” haplotype had strong linkage disequilibria with less risk of non-traumatic ONFH than the “CGC” wild-type. On the other hand, we detected no association between *MMP2* genetic variation and non-traumatic ONFH.

In *MMP2*, the SNPs are located on chromosome 16q13-q21; in *MMP9* they are located on chromosome 20q11.2-q13.1. *MMP9* SNP rs2274755 is located within an intron (boundary), which is consistent with it playing an important role in the development of non-traumatic ONFH. Jimenez-Morales et al. [6] reported that rs2274755 is associated with the risk for asthma in Mexican pediatric patients. Similar observations were made by Nakashima et al. in a Japanese population [26]. Rs2274755 has also been associated with myopia and refractive error as well as with the risk of steroid-induced ONFH in a population from northern China [19]. This is consistent with our results and may explain the similarity between steroid-induced and other forms of ONFH. On the other hand, a study of polyploidal choroidal vasculopathy and age-related macular degeneration showed no association between rs2274755 and those two diseases [27].

MMP-9 expression is stimulated by NF-κB ligand (RANKL), which is essential for osteoclastogenesis [28–30]. Fujisaki et al. [31] showed that RANKL induces MMP-9 production in osteoclasts, and that it stimulates bone resorption in the presence of interleukin-1α [31]. Thus osteoclast-related MMP-9 may associate with bone resorption in non-traumatic ONFH. The activity of MMPs is controlled in part by endogenous inhibitors, including the tissue inhibitors of metalloproteinases (TIMPs). A limitation of this study is that we did not examine whether *TIMP-2* and *TIMP-1* SNPs associated with ONFH susceptibility. Quantitative measurement of metabolism products in different groups would also enhance our research greatly. The samples that we collected were all from a Chinese Han population living in the north of China. There is the possibility of a type I error (false positive) could be made due to confounding factors.

In sum, we observed that the *MMP9* rs2274755 SNP associates with a lower risk of non-traumatic ONFH in a Han Chinese population. This suggests a relationship between MMP-9 and the risk of non-traumatic ONFH. We believe these results will encourage further studies into the function of MMPs and clarification of the mechanism by which *MMP9* SNPs affect susceptibility to non-traumatic ONFH.

## MATERIALS AND METHODS

### Study population

We conducted a case-control study of 585 non-traumatic ONFH patients and 507 controls matched for age and gender. The research was approved by the Ethical Committee of Zhengzhou Traditional Chinese Medicine Traumatology Hospital. All the participants were recruited between May 2014 and January 2016 from the Zhengzhou Traditional Chinese Medicine (TCM) Traumatology Hospital in Zhengzhou, Henan. The control participants were enrolled at the Zhengzhou Medical Center. This study adhered to the principles of the Declaration of Helsinki. All of the study participants provided informed consent and filled out a questionnaire gathering demographic information. All the control subjects were healthy individuals without osteonecrosis or other diseases.

### Inclusion and exclusion criteria

Patients were diagnosed with ONFH based on the following criteria [32]. (1) Clinical non-traumatic ONFH symptoms and signs, including hip pain with internal rotation limitation and sometimes with aching of the knee and thigh. Patients often had a history of corticosteroid use, alcoholism, bone marrow disease, infection, or decompression sickness. (2) A low-intensity band on T1-weighted images and “double-line sign” on T2-weighted images. (3) CT scan and pelvic radiographs showed demarcating sclerosis and a “crescent sign” at the femoral head. (4) Radionuclide bone scan had a “cold-in-hot” appearance. (5) Histological examination revealed evidence of trabecular and bone marrow necrosis. Non-traumatic ONFH was diagnosed when a patient exhibited two or more of these five criteria. Those who met the criteria for ONFH were selected. Patients diagnosed based on MRI but were without abnormalities on pelvic radiography were also included for our study. Patients were excluded it they had a history of direct trauma or if there were a possible combination of causes. Patients were also if they had a chronic metabolic disorder affecting the kidney, heart, or liver.

The control subjects were all healthy individuals without osteonecrosis and other diseases. The selection criteria for the controls were: (1) no history of hip pain; (2) no lesions on pelvic radiographs; (3) no history of corticosteroid usage or alcoholism; and (4) no relation to the enrolled patients.

### Genotyping

Genomic DNA was extracted from whole blood using an extraction kit (GoldMag) and stored at -20°C. The DNA concentration was measured using a NanoDrop 2000 spectrophotometer. All SNPs were >5%, which is the minor allele frequency in the Hap Map of the Chinese Han Beijing population. A total of eight SNPs have been detected in *MMP2 and MMP9*. For genotyping, we used Sequenom MassARRAY Assay Design 4.0 Software [33] and Multiplexed SNP Mass EXTEND assay [34].

### Statistical analysis

Statistical analyses were performed using Microsoft Excel and the SPSS 18.0 statistical package (SPSS, Chicago, IL). Values of p < 0.05 were considered statistically significant. Two-sided χ^2^ tests were used to calculate the genotype frequencies of the case and control individuals [35]. We assessed whether the genotype frequency of each SNP adhered to the Hardy-Weinberg equilibrium (HWE) using Fisher’s exact test. The genotype frequencies of cases and controls were compared using the χ^2^ test [36]. We determined odds ratios (ORs) and 95% confidence intervals (95%, CIs) using unconditional logistic regression analysis with adjustment for age and gender [37]. Finally, Haploview software (version 4.2) was used to estimate the pairwise linkage disequilibrium (LD).

## References

[1] Zhou X, Yishake M, Li J, Jiang L, Wu L, Liu R, Xu N (2015). Genetic susceptibility to prosthetic joint infection following total joint arthroplasty: a systematic review. Gene.

[2] Li Y, Wang Y, Guo Y, Wang Q, Ouyang Y, Cao Y, Jin T, Wang J (2016). OPG, RANKL polymorphisms are associated with alcohol-induced osteonecrosis of the femoral head in the north area of China population in men. Medicine (Baltimore).

[3] Wang JZ, Wang Y, Zhao Y, Li YZ, Sun MQ, Na RS, Jin TB, Yang XJ (2016). Polymorphisms of genes in the OPG/RANKL/RANK pathway in the Mongols of Inner Mongolia China: relationship to other populations. Int J Clin Exp Med.

[4] Wang Y, Cao YJ, Li YZ, Guo YC, Wang QJ, Yang M, Zhang N, Jin TB, Wang JZ (2015). Genetic association of the ApoB and ApoA1 gene polymorphisms with the risk for alcohol-induced osteonecrosis of femoral head. Int J Clin Exp Pathol.

[5] Banday MZ, Sameer AS, Mir AH, Mokhdomi TA, Chowdri NA, Haq E (2016). Matrix metalloproteinase (MMP) -2, -7 and -9 promoter polymorphisms in colorectal cancer in ethnic Kashmiri population - a case-control study and a mini review. Gene.

[6] Jimenez-Morales S, Martinez-Aguilar N, Gamboa-Becerra R, Jimenez-Ruiz JL, Lopez-Ley D, Lou H, Saldana-Alvarez Y, Dean M, Orozco L (2013). Polymorphisms in metalloproteinase-9 are associated with the risk for asthma in Mexican pediatric patients. Human Immunol.

[7] Amalinei C, Caruntu ID, Giusca SE, Balan RA (2010). Matrix metalloproteinases involvement in pathologic conditions. Rom J Morphol Embryol.

[8] Solli AI, Fadnes B, Winberg JO, Uhlin-Hansen L, Hadler-Olsen E (2013). Tissue- and cell-specific co-localization of intracellular gelatinolytic activity and matrix metalloproteinase 2. J Histochem Cytochem.

[9] Itoh T, Matsuda H, Tanioka M, Kuwabara K, Itohara S, Suzuki R (2002). The role of matrix metalloproteinase-2 and matrix metalloproteinase-9 in antibody-induced arthritis. J Immunol.

[10] Syggelos SA, Aletras AJ, Smirlaki I, Skandalis SS (2013). Extracellular matrix degradation and tissue remodeling in periprosthetic loosening and osteolysis: focus on matrix metalloproteinases, their endogenous tissue inhibitors, and the proteasome. Biomed Res Int.

[11] Hsu SM, Raine L, Fanger H (1981). The use of antiavidin antibody and avidin-biotin-peroxidase complex in immunoperoxidase technics. Am J Clin Pathol.

[12] Grassel S, Beckmann J, Rath B, Vogel M, Grifka J, Tingart M (2010). Expression profile of matrix metalloproteinase-2 and -9 and their endogenous tissue inhibitors in osteonecrotic femoral heads. Int J Mol Med.

[13] Vu TH, Shipley JM, Bergers G, Berger JE, Helms JA, Hanahan D, Shapiro SD, Senior RM, Werb Z (1998). MMP-9/gelatinase B is a key regulator of growth plate angiogenesis and apoptosis of hypertrophic chondrocytes. Cell.

[14] Murphy G, Knäuper V, Atkinson S, Butler G, English W, Hutton M, Stracke J, Clark I (2002). Matrix metalloproteinases in arthritic disease. Arthritis Res.

[15] Yoshihara Y, Nakamura H, Obata K, Yamada H, Hayakawa T, Fujikawa K, Okada Y (2000). Matrix metalloproteinases and tissue inhibitors of metalloproteinases in synovial fluids from patients with rheumatoid arthritis or osteoarthritis. Ann Rheum Dis.

[16] Wu HD, Bai X, Chen DM, Cao HY, Qin L (2013). Association of genetic polymorphisms in matrix metalloproteinase-9 and coronary artery disease in the Chinese Han population: a case-control study. Genet Test Mol Biomarkers.

[17] Zhang Y, Wang M, Zhang S (2016). Association of MMP-9 gene polymorphisms with glaucoma: a meta-analysis. Ophthalmic Res.

[18] Wang XL, Liu O, Qin YW, Zhang HJ, Lv Y (2014). Association of the polymorphisms of MMP-9 and TIMP-3 genes with thoracic aortic dissection in Chinese Han population. Acta Pharmacol Sin.

[19] Du J, Liu W, Jin T, Zhao Z, Bai R, Xue H, Chen J, Sun M, Zhang X, Wang G, Wang J (2016). A single-nucleotide polymorphism in MMP9 is associated with decreased risk of steroid-induced osteonecrosis of the femoral head. Oncotarget.

[20] Schache M, Baird PN (2012). Assessment of the association of matrix metalloproteinases with myopia, refractive error and ocular biometric measures in an Australian cohort. PLoS One.

[21] Liang J, Zhao T, Yang J, Li W, Zhang F, Zhang S, Huang Z, Lin R, Zhang X (2015). MMP-9 gene polymorphisms (rs3918242, rs3918254 and rs4810482) and the risk of psoriasis vulgaris: no evidence for associations in a Chinese Han population. Immunol Lett.

[22] Tsai EM, Wang YS, Lin CS, Lin WY, Hsi E, Wu MT, Juo SH (2013). A microRNA-520 mirSNP at the MMP2 gene influences susceptibility to endometriosis in Chinese women. J Hum Genet.

[23] Han DH, Kim SK, Kang S, Choe BK, Kim KS, Chung JH (2008). Matrix metallopeptidase 2 gene polymorphism is associated with obesity in Korean population. Korean J Physiol Pharmacol.

[24] Yu Y, Xie Z, Wang J, Chen C, Du S, Chen P, Li B, Jin T, Zhao H (2016). Single-nucleotide polymorphisms of MMP2 in MMP/TIMP pathways associated with the risk of alcohol-induced osteonecrosis of the femoral head in Chinese males: a case-control study. Medicine (Baltimore).

[25] Kim TH, Baek JI, Hong JM, Choi SJ, Lee HJ, Cho HJ, Park EK, Kim UK, Kim SY (2008). Significant association of SREBP-2 genetic polymorphisms with avascular necrosis in the Korean population. BMC Med Genet.

[26] Nakashima K, Hirota T, Obara K, Shimizu M, Doi S, Fujita K, Shirakawa T, Enomoto T, Yoshihara S, Ebisawa M (2006). A functional polymorphism in MMP-9 is associated with childhood atopic asthma. Biochem Biophys Res Commun.

[27] Zeng R, Zhang X, Wu K, Su Y, Wen F (2014). MMP9 gene polymorphism is not associated with polypoidal choroidal vasculopathy and neovascular age-related macular degeneration in a Chinese Han population. Ophthalmic Genet.

[28] Wittrant Y, Theoleyre S, Couillaud S, Dunstan C, Heymann D, Redini F (2003). Regulation of osteoclast protease expression by RANKL. Biochem Biophys Res Commun.

[29] Lorenzo JA, Pilbeam CC, Kalinowski JF, Hibbs MS (1992). Production of both 92- and 72-kDa gelatinases by bone cells. Matrix.

[30] Kusano K, Miyaura C, Inada M, Tamura T, Ito A, Nagase H, Kamoi K, Suda T (1998). Regulation of matrix metalloproteinases (MMP-2, -3, -9, and -13) by interleukin-1 and interleukin-6 in mouse calvaria: association of MMP induction with bone resorption. Endocrinology.

[31] Fujisaki K, Tanabe N, Suzuki N, Kawato T, Takeichi O, Tsuzukibashi O, Makimura M, Ito K, Maeno M (2007). Receptor activator of NF-kappa B ligand induces the expression of carbonic anhydrase II, cathepsin K, and matrix metalloproteinase-9 in osteoclast precursor RAW264.7 cells. Life Sci.

[32] Sugano N, Kubo T, Takaoka K, Ohzono K, Hotokebuchi T, Matsumoto T, Igarashi H, Ninomiya S (1999). Diagnostic criteria for non-traumatic osteonecrosis of the femoral head. A multicentre study. J Bone Joint Surg Br.

[33] Trembizki E, Smith H, Lahra MM, Chen M, Donovan B, Fairley CK, Guy R, Kaldor J, Regan D, Ward J, Nissen MD, Sloots TP, Whiley DM (2014). High-throughput informative single nucleotide polymorphism-based typing of Neisseria gonorrhoeae using the Sequenom MassARRAY iPLEX platform. J Antimicrob Chemother.

[34] Gabriel S, Ziaugra L, Tabbaa D (2009). SNP genotyping using the Sequenom MassARRAY iPLEX platform. Curr Protoc Hum Genet.

[35] Adamec C (1964). [Example of the use of the nonparametric test. Test x2 for comparison of 2 independent examples]. [Article in Czech]. Cesk Zdrav.

[36] Kochl S, Niederstatter H, Parson W (2005). DNA extraction and quantitation of forensic samples using the phenol-chloroform method and real-time PCR. Methods Mol Biol.

[37] Bland JM, Altman DG (2000). Statistics notes. The odds ratio. BMJ.

